# Considerations in the Design of Clinical Trials to Test Novel Entomological Approaches to Dengue Control

**DOI:** 10.1371/journal.pntd.0001937

**Published:** 2012-11-29

**Authors:** Marcel Wolbers, Immo Kleinschmidt, Cameron P. Simmons, Christl A. Donnelly

**Affiliations:** 1 Oxford University Clinical Research Unit, Hospital for Tropical Diseases, Ho Chi Minh City, Viet Nam; 2 Centre for Tropical Medicine, Nuffield Department of Medicine, University of Oxford, Oxford, United Kingdom; 3 MRC Tropical Epidemiology Group, Department of Infectious Disease Epidemiology, London School of Hygiene and Tropical Medicine, London, United Kingdom; 4 Medical Research Council Centre for Outbreak Analysis and Modelling, Department of Infectious Disease Epidemiology, School of Public Health, Imperial College London, London, United Kingdom; Mahidol University, Thailand

## Introduction

Dengue is the most important arboviral infection of humans. In endemic countries the scale of the dengue disease burden imparts an economic cost [1] and strains fragile health care systems. There are no licensed vaccines for prevention of dengue, and the public health response in endemic countries relies mostly on combating the principal mosquito vector, *Aedes aegypti*, via insecticides and breeding site removal. The sustained transmission of dengue in endemic settings together with its increasing global footprint indicates existing disease control strategies have been unsuccessful [2].

Novel vector control approaches to limit dengue virus (DENV) transmission include release of *Ae. aegypti* that carry transgenes that result in highly penetrant, dominant, late-acting, female-specific lethality [3]. In field cage experiments, the release of such mosquitoes in sufficient numbers results in eradication of the mosquito population [4]. Another strategy involves embryonic introduction of the obligate intracellular insect bacterium, *Wolbachia*, into strains of *Ae. aegypti* [5]. Strikingly, *Wolbachia*-infected *Ae. aegypti* are partially resistant to infection with DENV [6], and by virtue of the intrinsic capacity of some strains of *Wolbachia* to invade insect populations [6], [7], there is the prospect of achieving widespread biological resistance to DENV amongst *Ae. aegypti* populations. The life-shortening impact of some Wolbachia strains could also contribute to reductions in disease transmission [5]. The first entomological field trials of mosquitoes infected with *Wolbachia* (wMel and wMelPop strains) have now been successfully carried out in Cairns, Australia and have demonstrated that *Wolbachia* can establish itself at very high prevalence in field populations of *Ae. aegypti* [7]. However, the prospects of demonstrating reduction in DENV transmission in Cairns are slim given the episodic, imported nature of dengue outbreaks in this region.

A critical challenge for all entomological approaches to control of vector-borne disease is how best to demonstrate efficacy in reducing disease transmission [8]. In principal, the high force of infection in dengue endemic countries should assist an evidence-gathering approach to this challenge. However, a feature of dengue epidemiology is that it is spatially and temporally heterogeneous [9]–[11]. Thus oscillations in disease incidence over time are common for a given region of transmission, and within each region it is common for focal “hot spots” of transmission to exist [3]. This heterogeneity in transmission means that uncontrolled observational studies of dengue transmission in a community where, for example, *Wolbachia*-infected *Ae. aegypti* have been released could take many years or decades to yield evidence that is suggestive of a benefit. Equally, the heterogeneity of dengue transmission poses challenges to traditional clinical trial approaches, as does the non-stationary nature of mosquito populations [8]. Here we review design and statistical considerations relevant to the conduct of clinical trials of these novel interventions and the practical challenges posed by the epidemiology of dengue in endemic settings. Whilst our discussion of trial design is focused on *Wolbachia*-infected *Ae. aegypti*, it is also relevant to other vector control interventions, such as genetically engineered male mosquitoes carrying a dominant lethal gene [4], insecticide-impregnated nets [12], or larvacides [13].

## Methods

Cluster randomised trials (CRTs) are the gold standard design to provide evidence on the efficacy of an intervention that has community-wide impact [14]. Cluster formation is a crucial aspect of the design of a CRT and requires prior mapping of the study area with respect to dengue sero-prevalence, demographics, and information on movement of individuals. Experience from the Cairns (Australia) release shows that it is feasible to achieve a prevalence of Wolbachia infection in *A. aegypti* mosquitoes of nearly 100% in treatment clusters within 6 months after first release [7]. Clusters need to be sufficiently geographically separated to ensure that *A. aegypti* mosquitoes present in control clusters remain virtually free of *Wolbachia* for the entire study period.

We consider the incidence of DENV-seroconversions during a trial as a suitable primary endpoint and DENV-naïve children aged 2–5 years living in each cluster as an optimal “sentinel” cohort for serological surveillance. Young children are less likely to spend substantial periods of time outside of their residence and local community (and hence outside of the “treatment umbrella”) than more mobile older children and adults. In addition, DENV-prevalence in older children is higher and those remaining naïve and hence eligible for the study are potentially less representative of the full population (for example, for socio-economic reasons).

Two alternative designs are considered. The first is the classical parallel two-armed cluster randomised trial (PCRT) in which each recruited cluster is randomised to intervention or control, and the intervention is implemented simultaneously across the relevant clusters. Thus the control clusters provide contemporaneous controls for the intervention clusters. The other design considered is a stepped wedge cluster randomised trial (SWCRT) in which each cluster is assigned to the control treatment initially and clusters are subsequently crossed-over to the intervention in a random selection at fixed time points until eventually all clusters are under treatment [15], [16]. As dengue is a seasonal disease, selected cross-over time points should reflect this. As an example, for a 3-year study period, the SWCRT has: all clusters as controls for year 1; half of the clusters as controls and half as intervention, randomly selected, for year 2; and all clusters on intervention in year 3. Diagrams of both designs are provided in Text S1.

SWCRTs have been most frequently used for evaluating interventions during routine implementation such as the evaluation of a vaccine on the community level following a successful individual randomised trial. From a logistic perspective, they are attractive, because the intervention can be rolled out in a step-wise fashion and evaluated. As clusters are their own controls, SWCRTs are less sensitive to between-cluster variation and thus might require a lower sample size compared to parallel designs [15]. However, strong temporal effects may greatly reduce the precision of estimates as all clusters start out in the control arm and end as intervention clusters. Secular trends of dengue during the study period could confound the treatment effect causing bias. SWCRTs are less flexible for trial adaptations such as an extension of the follow-up period if the observed DENV-incidence is lower than expected, as all clusters have already crossed-over to the intervention at this time point.

Cluster size and cluster separation are important considerations in the design of all CRTs, but they require particular attention in trials of vector control interventions, for which entomological and community considerations need be taken into account. Entomological considerations include the dispersal of *Wolbachia*-infected mosquitoes to ensure a persistent and homogenous effect in treatment clusters without undue contamination into untreated clusters that serve as controls. For dengue trials community considerations include the extent of daily movement within and between clusters that the surveillance cohorts are likely to undertake; if the clusters are too small this movement may be excessive, and cause further reduction in any treatment effect. Thus, data on movement patterns of children eligible to join the surveillance cohort together with more information on the limits of spatial dispersal of *Wolbachia*-infected mosquitoes are essential before the cluster formation stage of any trial. An approach that is widely adopted in CRTs is the so-called “fried-egg” design [14], in which the whole cluster receives the allocated treatment but only the inner area of the cluster (the “egg-yolk”) is used for surveillance since the treatment effect in this inner area is less affected by spill-over from neighbouring clusters that may be in the opposite treatment arm. We would therefore suggest that the surveillance cohort in each cluster be drawn from this inner area of each cluster.

## Sample Size Requirements of a CRT

Sample size requirements for CRTs of a *Wolbachia* intervention (or other community-based intervention) depend critically on the size of the intervention effect and on both the magnitude and the variability (temporal and spatial) of seroconversion rates between clusters. To assess this variability in an example, we used published data from 12 primary schools in Kamphaeng Phet, Thailand, followed over a 3-year period [10] where the overall yearly DENV infection incidences were 7.9%, 6.5%, and 2.2%.

A mixed-effects Poisson-regression model fitted to these data gave coefficients of variation (cv, i.e., SD/mean) for yearly DENV infection incidence of 0.27 for between-school variation, 0.57 for annual variation, and 0.85 for residual variation (i.e., variation that cannot be explained by systematic spatial or temporal variation, respectively, and corresponds to localized school and year specific variation). A detailed description of the model used to derive these coefficients of variation can be found in Text S1. The overall between-school coefficient of variation over the 3-year period was 0.52. The same model fit to data from 43 villages in Cambodia [9], also showed that temporal and residual variation are more pronounced than spatial variation (unpublished data).

We then used the incidence and variability data reported above to simulate hypothetical PCRT and SWCRT trials. Additional assumptions for the trial simulations were a study duration of 3 years and a surveillance cohort of 100 children in each cluster. We varied the intervention effect between a 40% and an 80% decrease of DENV seroconversion in intervention clusters compared to controls. Allowing for the fact that some children in intervention clusters will experience infections outside of the intervention area, we regard an effect of a 50%–60% reduction as realistic in our target population. Details regarding the set-up of the simulation study and the statistical analysis of simulated trials are provided in Text S1.

## Results

**Figure 1:**
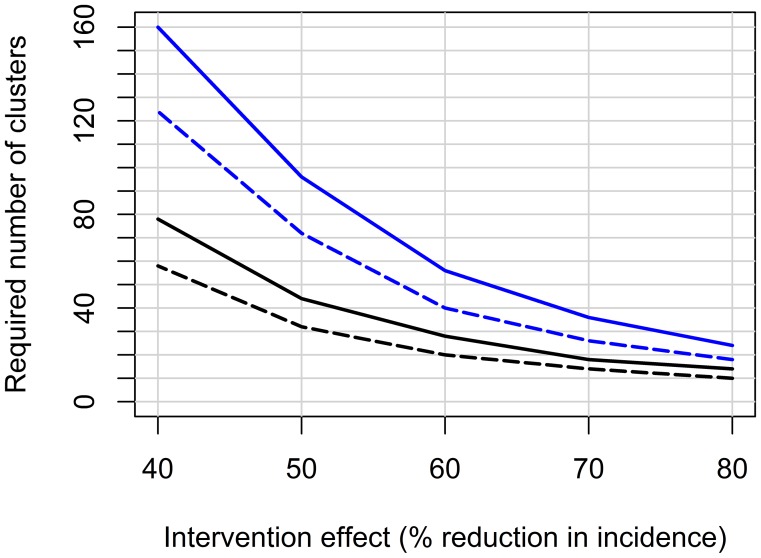
Sample size estimates for a PCRT or a SWCRT. Total number of clusters required for a PCRT (black lines) or a SWCRT (blue lines) depending on the size of the intervention effect. Solid lines correspond to 90% power, dashed lines to 80% power. Simulations are based on parameters determined from the Kamphaeng Phet dengue cohort (Thailand) (described in [10]) with three time periods each of 1-year duration, a surveillance cohort of 100 children in each cluster, and a two-sided significance level of 5%.

Sample size requirements for the two designs and for varying treatment effects are shown in Figure 1 and requirements for several alternative scenarios are given in Text S1. The required total sample sizes to detect a 60% or 50% reduction of dengue in the intervention arm with 80% power were 20 or 32 clusters, respectively, for a PCRT compared to 40 or 72 clusters for a SWCRT. The SWCRT design generally required substantially higher sample sizes except in the unrealistic situation of spatial but no temporal or residual variation.

## Conclusions

A parallel cluster-randomised trial is the design of choice for testing novel entomological methods of dengue control. Under realistic assumptions we show it to require a substantially lower sample size than a stepped wedge design. Sample size requirements for a parallel design are relatively modest; our example gave a minimum sample size of 20 clusters (ten per study arm) with each cluster providing 100 person-years of follow-up per year and a follow-up duration of 3 years. Although careful planning and substantial funding are required to run such a trial, the benefits of having a robust evidence-base from which to promote programmatic roll-out and/or further optimisation of the strategy should prove invaluable.

## Supporting Information

Text S1 Statistical appendix containing: (1) a diagram of a parallel two-arm cluster randomised trial (PCRT) and a stepped wedge cluster randomised trial (SWCRT), (2) details regarding the determination of coefficients of variation for the Thailand data, and (3) details regarding the simulation study to compare PCRT versus SWCRT designs.
(DOCX)
