# Supplementary material for: Considerations in the Design of Clinical Trials to Test Novel Entomological Approaches to Dengue Control
Source: PLoS Negl Trop Dis. 2012 Nov 29;6(11):e1937. doi: 10.1371/journal.pntd.0001937 (PMC3510076; doi:10.1371/journal.pntd.0001937)
Supplement: Text S1 — Statistical appendix containing: (1) a diagram of a parallel two-arm cluster randomised trial (PCRT) and a stepped wedge cluster randomised trial (SWCRT), (2) details regarding the determination of coefficients of variation for the Thailand data, and (3) details regarding the simulation study to compare PCRT versus SWCRT designs. (DOCX) [file pntd.0001937.s001.docx]

# Statistical Appendix for “Considerations in the design of clinical trials to test novel entomological approaches to dengue control”

Figure 1. Diagram of a parallel two-arm Cluster Randomised Trial (PCRT) and a stepped wedge Cluster Randomised Trial (SWCRT) with 12 clusters and 4 time periods.

a) PCRT

| Cluster  No. | 1 | 2 | 3 | 4 | 5 | 6 | 7 | 8 | 9 | 10 | 11 | 12 |
| --- | --- | --- | --- | --- | --- | --- | --- | --- | --- | --- | --- | --- |
| Year 1 | C | C | C | C | C | C | I | I | I | I | I | I |
| Year 2 | C | C | C | C | C | C | I | I | I | I | I | I |
| Year 3 | C | C | C | C | C | C | I | I | I | I | I | I |
| Year 4 | C | C | C | C | C | C | I | I | I | I | I | I |

b) SWCRT

| Cluster  No. | 1 | 2 | 3 | 4 | 5 | 6 | 7 | 8 | 9 | 10 | 11 | 12 |
| --- | --- | --- | --- | --- | --- | --- | --- | --- | --- | --- | --- | --- |
| Year 1 | C | C | C | C | C | C | C | C | C | C | C | C |
| Year 2 | I | I | I | I | C | C | C | C | C | C | C | C |
| Year 3 | I | I | I | I | I | I | I | I | C | C | C | C |
| Year 4 | I | I | I | I | I | I | I | I | I | I | I | I |

C=control, I=intervention

## Determination of coefficients of variation for the Thailand data

Calculations are based on published data from 12 primary schools in Kamphaeng Phet, Thailand, followed over a 3-year period [[10](#_ENREF_10)] where an average yearly dengue incidence of 4.8% was observed.

We used the following mixed effects Poisson regression model to estimate the extent of temporal, spatial, and residual variation at location i and time period j:

log(number of seroconversions)_ij_ ~ μ+λ_i_+ν_j_+ε_ij_+offset(log(persontime))
with λ_i_~N(0,σ_λ_^2^), ν_j_~N(0,σ_ν_^2^), ν_j_~N(0,σ_ε_^2^) (1)

In this model, the observed number of dengue seroconversions is the outcome, the observed log-person- years of follow-up is an offset, μ corresponds to the “null” log-incidence (assuming no random effects), and the variance components σ_λ_^2^, σ_ν_^2^ and σ_ε_^2^ determine the amount of spatial, temporal, and residual variation, respectively. We fitted this model with the R-package lme4 version 0.999375-42 to the published data and obtained estimates μ=-3.40, σ_λ_= 0.27, σ_ν_ =0.53, and σ_ε_= 0.74.

Model (1) specifies a log-normal distribution for the expected number of dengue seroconversions in each location and time period. The coefficient of variation of a lognormal distribution is given by $cv=\sqrt{\exp\left( \sigma^{2} \right)-1}$ where σ^2^ is the variance of the log-transformed data. Thus, for the above example, the coefficient of variation corresponding to spatial variation is given by $cv=\sqrt{\exp\left( {0.27}^{2} \right)-1}=0.27$ (as mentioned in the main manuscript) and the corresponding coefficients of variation for temporal and residual variation are calculated in the same way.

## Simulation study

### Simulation of scenarios

We simulated artificial clinical trials to determine required sample sizes and to compare the PCRT to the SWCRT. The basis for the simulation was model (1) from above with the inclusion of an additional intervention effect. For example, an intervention effect of log(0.5) corresponds to a 50% reduction in dengue incidence. Thus, the simulation first simulated expected numbers of seroconversions at location i and time j according to (1) and the hypothesized intervention effect and subsequently simulated the observed number of dengue seroconversions according to a Poisson distribution with this expected value.

The base scenario included the parameters determined from the Thailand data summarized above, a 50% reduction in dengue incidence due to treatment, 3 time periods, a cluster size resulting in the observation of 100 child-years per annum (period), a two-sided significance level of 5%, and a target power of 90%. For this scenario, and various alternative scenarios, the required number of clusters for a PCRT and a SWCRT to satisfy these criteria were determined.

### Analysis

The analysis for the PCRT was based on the aggregated data which contained the total number of dengue seroconversions in a cluster during the entire follow-up period as outcome. We used a Poisson regression with a quasi-likelihood correction for overdispersion. Pseudo-code for fitting such a model with the statistical software R, is

glm(count~trt+offset(log(persontime)),data=data.aggr,family=quasipoisson())

where data.aggr corresponds to the aggregated data.

This analysis for a PCRT is simple and robust to model misspecification. However, an alternative analysis based on raw (non-aggregated) data similar to the analysis of the SWCRT outlined below might gain additional power.

The analysis for the SWCRT was a mixed effects Poisson regression of yearly numbers of dengue seroconversions with fixed treatment and period effects and random effects for the location (cluster) and residual (spatio-temporal) variation. Pseudo-code for fitting such a model with the statistical software R and the companion package lme4 is

glmer(count~trt+period+(1|cluster)+(1|cluster.period),
 offset=log(persontime),data=data,family=poisson())

### Other simulation conventions

Sample size calculations were based on simulation (as outlined above) and simulation of 1000 trials for each parameter setting. The number of clusters was successively increased until the target power was achieved.

### Simulation results

Figure 1 of the main text shows required total number of clusters for a PCRT or SWRT depending on the size of the intervention effect and a target power of 80% or 90% at the two sided 5% significance level. The simulations are based on parameters determined from the Thailand data, 3 time periods, and a cluster size resulting in the observation of 100 child-years of follow-up per cluster and annum (period).

Table 1 below shows required total numbers of clusters for PCRT and SWCRT designs for several additional scenarios.

Table 1. Required total number of clusters for a PCRT or a SWCRT, respectively, for various scenarios. All scenarios are based on an assumed intervention effect corresponding to a 50% reduction in dengue incidence, a two-sided significance level of 5%, and a power of 90%.

| Scenario description | “Null” log-incidence  Μ | Spatial variation  σ_λ_ | Temporal variation  σ_ν_ | Residual variation  σ_ε_ | Number of time periods | Child-years of follow-up per cluster and period | Required number of clusters for PCRT | Required number of clusters for SWCRT |
| --- | --- | --- | --- | --- | --- | --- | --- | --- |
| Base scenario | -3.40 | 0.27 | 0.53 | 0.74 | 3 | 100 | 44 | 96 |
| 5 instead of 3 periods | -3.40 | 0.27 | 0.53 | 0.74 | **5** | 100 | 32 | 40 |
| Less follow-up per cluster | -3.40 | 0.27 | 0.53 | 0.74 | 3 | **50** | 54 | 132 |
| More follow-up per cluster | -3.40 | 0.27 | 0.53 | 0.74 | 3 | **200** | 40 | 78 |
| 2-fold higher incidence | **-2.71** | 0.27 | 0.53 | 0.74 | 3 | 100 | 40 | 76 |
| 2-fold lower variation | -3.40 | **0.135** | **0.265** | **0.37** | 3 | 100 | 20 | 52 |
| Only spatial and temporal variation^#^ | -3.23 | 0.51 | 0.55 | - | 3 | 100 | 34 | 44 |
| Only spatial variation^#^ | -3.06 | 0.49 | - | - | 3 | 100 | 34 | 32 |
| Only temporal variation^#^ | -3.02 | - | 0.55 | - | 3 | 100 | 12 | 32 |

^#^ Parameters for μ, σ_λ_, σ_ν_ , and σ_ε_ are based on maximum likelihood fits of the reduced models to the Thailand data. All reduced models fitted the data much worse in terms of Akaike’s information criterion (AIC) than the full model of the base scenario.
